# The Sex Ratio at Birth for 5,338,853 Deliveries in China from 2012 to 2015: A Facility-Based Study

**DOI:** 10.1371/journal.pone.0167575

**Published:** 2016-12-12

**Authors:** Yan Huang, Wen Tang, Yi Mu, Xiaohong Li, Zheng Liu, Yanping Wang, Mingrong Li, Qi Li, Li Dai, Juan Liang, Jun Zhu

**Affiliations:** 1 Nursing Department, West China Second University Hospital, Sichuan University, Chengdu, Sichuan, China; 2 National Office for Maternal and Child Health Surveillance of China, West China Second University Hospital, Key Laboratory of Birth Defects and Related Diseases of Women and Children, Ministry of Education, Sichuan University, Chengdu, Sichuan, China; Shanghai Jiaotong University School of Medicine Xinhua Hospital, CHINA

## Abstract

**Objective:**

The accuracy of a population-based sex ratio at birth (SRB) in China has long been questioned. To depict a more accurate profile, the present study used data from a national surveillance system for health facility births to explore the characteristics of SRB in China.

**Methods:**

Data from China’s National Maternal Near Miss Surveillance System between 2012 and 2015 were used. We restricted the analysis to live births of ≥28 completed gestational weeks or ≥1000 g birth weight. The strength of association between obstetric characteristics and SRB was examined using logistic regression, taking into account the sampling strategy and clustering of births within health facilities.

**Results:**

There were 2,785,513 boys and 2,549,269 girls born alive between 2012 and 2015 in 441 health facilities. The SRB was 111.04 in 2012, 110.16 in 2013, 108.79 in 2014, and 109.53 in 2015. The SRB was high in the eastern region, especially in rural areas. The SRBs increased with mother’s age and decreased with mother’s education. The SRB in women who were pregnant for the first time was 104.30. The SRB in primipara was normal (104.35), but it was extremely high in non-primipara, especially for women with three or more parities (141.76); only 5.26% of live births fell within this group. The SRBs increased significantly by the number of parities, especially in the rural areas of the central region. After adjustment for sociodemographic factors, women with three or more parities were 1.39 (95% CI 1.34, 1.43) times more likely to give birth to a boy compared with primiparae who were pregnant for the first time.

**Conclusion:**

Our analysis suggests that the SRB was lower than what was reported officially but higher than normal. The government should keep strengthening supervision to prevent sex-selection, especially in the wake of the two-child policy implemented in 2015.

## Introduction

Sex ratio at birth (SRB) is defined as the number of boys born per 100 girls[1]. According to the genetics of sex determination, the SRB should theoretically be equal to 100, but in a large population, the average SRB ranges between 103 and 107[2]. The slight shift to a preponderance of boys by the time of delivery has been explored in the past few decades, but it remains a biologic mystery. The limited evidence available indicated that the Y sperm may have a greater chance of success in the female reproductive tract or in fertilization of the ovum[3]. The SRB is considered to be consistently stable in human populations, and any remarkable deviation from this ‘global average’ of 105±2, or ‘imbalanced sex ratio’, is attributed to sex-selective under-reporting, sex-selective abortion, or other man-made factors[4].

An imbalanced sex ratio has been found in a number of Asian countries, including India, Vietnam, Korea and China[2, 5–8], where the tradition of son-preference still prevails. As the most populous countries in the world, China’s SRB has been reported as a serious social problem since the 1980s. Data from national census shows that the SRB was 108.94 in 1982, 111.87 in 1990, 116.90 in 2000 and 117.94 in 2010 [9]. The highest SRB of 121.18 was reported in 2004 by the National Bureau of Statistics of China[10]. China’s nationwide population census is implemented by census enumerators through door-to-door household surveys. In principle, personal data shall be declared by the parties directly. But not everyone can make self-declaration, such as infants and young children. In the actual survey, a person familiar with the situation is appointed by the household to declare family data according to the facts. China is one of the countries that son-preference has been around for a number of years[11]. However, the national one-child policy, which intended to restrict the rapid growth of China’s population, pushed families to prefer the birth of boys. As a result, girls were frequently concealed or omitted from declaration or it was reported that girls had died[12, 13]. This was done to escape the punishment by the government or to obtain permission for another child. Thus, it is generally accepted that the number of registered people is not exactly accurate in the census. In addition, China is a populous country, and its increasing floating population (nearly 2.53 million in 2014)[14], adds to the difficulty of checking registering data, so the number of these unregistered girls who have not been investigated has remained a mystery[12, 15].

The increasing SRB trend in China, if it continues to last for a long time and covers a wide range of regions may be hazardous. Social problems may arise as a result, such as the “marriage squeeze” (the imbalance between marriageable males and females entails that some males or females will be unable to choose their spouse according to the generally accepted criteria, and a number of people will fail to marry at all)[16], “mercenary marriage” (maimaihunyin, which refers to a marriage arranged by a third party, including parents or a kidnapper, who, in order to obtain money or property, illegally force someone to marry[17]), the trafficking of women and children, sexual crime, and fewer opportunities for women development[18–22]. These problems will ultimately endanger the stable development of China in the long run.

Being aware of SRB’s potential short-term and long-term effects, the Chinese government has taken a number of prevention and intervention measures targeting the SRB, including a series of prohibitive laws, policies and regulations to forbid prenatal diagnosis of sex for non-medical reasons and sex-selective abortion for non-medical reasons. A nationwide campaign called “Care for Girls” has been running since 2005 to tackle the problem of prenatal diagnosis of sex for non-medical reasons and sex-selective abortion for non-medical reasons[2]. In recent years, the Chinese government has developed social security effectively and the number of people covered by basic insurance has increased rapidly since 2009[23]. Such measures have helped to offset the son-preference that sustains the traditional belief of “having a son to provide for old age”. In addition, more generous family planning policies were established from 2013. However, in 2015, the SRB from official reports was 113.51, still higher than normal[24].

In previous studies, the data used to calculate the SRB in China were mostly from Chinese census or population sampling investigations which are both population based and generally biased[12, 15]. Compared with population investigations, hospital records have been more in line with the actual sex of live births because doctors do not change the record of baby sex or conceal the birth of any baby. Therefore, in China where in-hospital delivery reaches 99.5% in 2014[25], hospital-based data are more accurate and reliable. In this study we used facility-based data representative of all facility births in China to calculate the SRB in order to compare it with official statistics, to explore regional and demographic variations in SRBs and to assess the effectiveness of the nationwide "Care for Girls" campaign.

## Materials and Methods

### Data

This study used the data from China’s National Maternal Near Miss Surveillance System (NMNMSS) between Jan. 1^st^, 2012, and Dec. 31^st^, 2015. The NMNMSS was established in Oct. 2010[26] in health facilities whose annual deliveries were more than 1000 and which were selected from 326 districts or counties in 30 provinces to surveillance national maternal deaths and maternal near misses. Because some districts or counties did not have hospitals with the required number of births, especially in rural areas, large hospitals in urban districts were oversampled, particularly in central and western regions (S1 Table)[27].

In China, the birth has been defined as a birth at no less than 28 completed weeks of gestation (if the gestational age is missing, the birth weight at no less than 1000 g is used). In our analysis, therefore, we restricted live births to the births of 28 or more completed weeks of gestation or 1000 g or heavier birthweight. For each pregnant woman at the surveillance health facilities, and for each postpartum woman who was admitted to obstetrics departments of the facilities within 42 days, the information from admission to discharge, including maternal characteristics and pregnancy outcomes, were prospectively collected.

In all 441 facilities, doctors responsible for patient care were trained to collect data prospectively from admission to discharge and to complete a specially designed data collection form for each woman. Data were collected for sociodemographic characteristics, obstetric history, method of delivery, pregnancy outcome, sex of the baby, and birth weight et. Data were entered into a web-based data management system and summarized at the National Office for Maternal and Child Health Surveillance of China. Quality control was conducted regularly[27]. All information that can be used to identify individual women are blocked or invisible to us.

The NMNMSS was approved by the Ethics Committee of West China Second University Hospital, Sichuan University, China (Protocol ID, 2012008; Date of approval, 03 Mar 2012).

### Statistical Analysis

First, the time trends and the geographic variations of SRB in China were examined. The sociodemographic characteristics of SRB were also explored using the same approach. Considering that the SRBs were related to the number of pregnancies and parities, we examined the association between SRB and these numbers using logistic regression by taking into account the clustering of births within facilities. On the one hand, large hospitals in urban districts were oversampled; on the other hand, data on the distribution of hospitals’ deliveries in each region and urban/rural area were unable to obtain. As a result, we used a sampling distribution by weighing the data against the probability of each individual being included in each region and rural/urban area (S1 Table). Crude odds ratios (ORs) with 95% confidence intervals (CI) and OR adjusting for sociodemographic characteristics were both reported. Statistical analyses were conducted using SAS 9.4 software.

## Results

In China, there were 5,338,853 live births at a gestational age of 28 or more completed weeks’ or a weight of 1000g or more in 441 health facilities between 2012 and 2015. Of the 5,338,853 live births, females accounted for 47.75% and males accounted for 52.17%, resulting in a SRB of 109.86. Between 2012 and 2014, the SRB dropped from 111.04 to 108.79. In 2015, the SRB increased slightly, reaching 109.53 (Table 1).

**Table 1:** Time trends and geographical variations of SRB in China (2012 to 2015).

		Number of live births	Female		Male		SRB	Adjusted SRB*
			Number	Percentage	Number	Percentage		
**Year**								
	**2012**	1318173 (24.69%)	625943	47.49	691570	52.46	110.48	111.04
	**2013**	1286906 (24.10%)	614090	47.72	672065	52.22	109.44	110.16
	**2014**	1465833 (27.46%)	703510	47.99	760630	51.89	108.12	108.79
	**2015**	1267941 (23.75%)	605726	47.77	661248	52.15	109.17	109.53
**Region**								
	**East**	1564536 (29.30%)	743910	47.55	817836	52.27	109.94	110.67
	**Central**	2121681 (39.74%)	1010287	47.62	1110657	52.35	109.93	109.91
	**West**	1652636 (30.95%)	795072	48.11	857020	51.86	107.79	108.85
**Area**								
	**East-Rural**	588403 (11.02%)	274971	46.73	313329	53.25	113.95	113.95
	**East-Urban**	976133 (18.28%)	468939	48.04	504507	51.68	107.58	107.58
	**Central-Rural**	1060638 (19.87%)	505365	47.65	555126	52.34	109.85	109.85
	**Central-Urban**	1061043 (19.87%)	504922	47.59	555531	52.36	110.02	110.02
	**West-Rural**	658306 (12.33%)	312719	47.50	345476	52.48	110.47	110.47
	**West-Urban**	994330 (18.62%)	482353	48.51	511544	51.45	106.05	106.05
**All**		5338853 (100.00%)	2549269	47.75	2785513	52.17	109.27	109.86

In the period 2012 to 2015, geographical variations in SRB were found (Table 1). The SRB in eastern China was 110.67, which was higher than that in central China (109.91) and western China (108.85). In all three regions, we found that the SRB in the urban areas of eastern China was 107.58, which was lower than that in rural areas (113.95). In central China, the SRB in urban areas (110.02) was slightly higher than that in rural areas (109.85). In western China, the SRB in urban areas (106.05) was lower than that in rural areas (110.47).

The sociodemographic and obstetric characteristics of SRB in China were summarized (Table 2). Only approximately 1.5% women were unmarried, but the SRB for the unmarried (109.01) was similar to that for the married (109.87). The SRB increased with the mother's age. The SRB for mothers aged 20 years or below was 105.69, which for mothers of 30 or more years exceeded 112, and that for 40 years or above reached 113.23. Nearly one-third of women attended college or higher education, for whom the SRB was the lowest, reaching 105.98. Approximately 43% of women were pregnant for the first time, and the SRB was 104.30, lower than that for mothers with their third or more pregnancy (117.99). Parity was also found to be correlated with the SRB. In primipara, the SRB was 104.35, and that for three or more parities was 141.76.

**Table 2:** The sociodemographic characteristics of SRBs in China (2012 to 2015).

		Number of live births	Female		Male		SRB	Adjusted SRB*
			Number	Percentage	Number	Percentage		
**Marital status**								
	**Unmarried**	79606 (1.49%)	38111	47.87	41431	52.05	108.71	109.01
	**Married**	5258078 (98.49%)	2510658	47.75	2743429	52.18	109.27	109.87
	**Missing**	1169 (0.02%)	500	42.77	653	55.86	130.60	127.53
**Mother's age**								
	**<20**	123992 (2.32%)	60163	48.52	63761	51.42	105.98	105.69
	**20~**	1095844 (20.53%)	527942	48.18	567362	51.77	107.47	107.48
	**25~**	2201678 (41.24%)	1053697	47.86	1146392	52.07	108.80	109.70
	**30~**	1199312 (22.46%)	567476	47.32	630717	52.59	111.14	112.29
	**35~**	416138 (7.79%)	195794	47.05	219873	52.84	112.30	113.11
	**40~**	102157 (1.91%)	47941	46.93	54100	52.96	112.85	113.23
	**Missing**	199732 (3.74%)	96256	48.19	103308	51.72	107.33	108.00
**Mother's education**								
	**College or higher**	1661627 (31.12%)	807544	48.60	852377	51.30	105.55	105.98
	**High school**	1422386 (26.64%)	678632	47.71	742422	52.20	109.40	109.86
	**Middle school**	1906370 (35.71%)	898666	47.14	1006992	52.82	112.05	111.98
	**Primary school**	193821 (3.63%)	91139	47.02	102579	52.92	112.55	112.35
	**Illiteracy**	30646 (0.57%)	14541	47.45	16081	52.47	110.59	111.44
	**Missing**	124003 (2.32%)	58747	47.38	65062	52.47	110.75	109.98
**Number of pregnancies**								
	**1**	2313376 (43.33%)	1133095	48.98	1178372	50.94	104.00	104.30
	**2**	1603104 (30.03%)	761566	47.51	840419	52.42	110.35	111.06
	**≥3**	1419916 (26.60%)	653440	46.02	765444	53.91	117.14	117.99
	**Missing**	2457 (0.05%)	1168	47.54	1278	52.01	109.42	107.12
**Parity**								
	**1**	3309593 (61.99%)	1620256	48.96	1686593	50.96	104.09	104.35
	**2**	1720786 (32.33%)	799564	46.47	920197	53.48	115.09	115.20
	**≥3**	280948 (5.26%)	115907	41.26	164823	58.67	142.20	141.76
	**Missing**	31161 (0.58%)	15204	48.79	15866	50.92	104.35	104.53
**All**		5338853 (100.00%)	2549269	47.75	2785513	52.17	109.27	109.86

*Adjusted for sampling distribution of population.

According to Table 2, we can see a woman’s number of pregnancies and parities were both strongly related with SRB, especially for parities. To know more about this relationship, we explored the association between SRB and the number of pregnancies and parities (Table 3). We found that the SRBs of women with different orders of pregnancy in primipara were approximately 104, but as the number of parities increased, the SRBs increased accordingly, reaching 141.76 in women with three or more parities. The same trends were also observed in different regions of China, although the SRBs in the western region increased by a relatively small margin with increasing number of parities compared with the eastern and central regions. When examining the difference in SRBs between urban and rural areas, we found that the SRBs were higher in the east-rural than east-urban regions regardless of how many pregnancies and parities a woman had. Nevertheless, the SRBs of the urban areas were higher than those of the rural areas in the central region. In west region, the SRB in women with three or more parities was higher in the urban areas than in the rural areas. In women with three or more parities, the SRB of central-urban areas was higher (162.08) than any other area in China.

**Table 3:** The SRB in each order of pregnancy and parity in China (2012–2015).

Variables			Parity		
			1	2	≥3
**East**					
	**Number of pregnancies**	**1**	104.94	-	-
		**2**	105.10	119.74	-
		**≥3**	104.35	116.56	151.38
**Central**					
	**Number of pregnancies**	**1**	104.01	-	-
		**2**	104.12	115.71	-
		**≥3**	106.60	113.44	150.82
**West**					
	**Number of pregnancies**	**1**	103.98	-	-
		**2**	103.24	112.25	-
		**≥3**	103.75	113.84	128.55
**East-Urban**					
	**Number of pregnancies**	**1**	103.69	-	-
		**2**	105.20	115.69	-
		**≥3**	103.89	114.32	146.62
**East-Rural**					
	**Number of pregnancies**	**1**	106.57	-	-
		**2**	104.94	122.09	-
		**≥3**	105.09	118.34	153.48
**Central-Urban**					
	**Number of pregnancies**	**1**	105.16	-	-
		**2**	105.09	118.32	-
		**≥3**	106.19	118.07	162.08
**Central-Rural**					
	**Number of pregnancies**	**1**	103.32	-	-
		**2**	103.12	115.09	-
		**≥3**	107.17	110.99	147.86
**West-Urban**					
	**Number of pregnancies**	**1**	101.24	-	-
		**2**	102.34	111.83	-
		**≥3**	102.97	112.61	132.37
**West-Rural**					
	**Number of pregnancies**	**1**	105.73	-	-
		**2**	104.17	112.36	-
		**≥3**	104.88	114.59	127.48
**All**					
	**Number of pregnancies**	**1**	104.32	-	-
		**2**	104.21	115.72	-
		**≥3**	104.90	114.58	141.76

All SRBs were adjusted for the sampling distribution of the population.

The association between the number of pregnancies and parities and SRB adjustment for area, mother's education, and mother’s age was also summarized and is shown in Table 4. In primipara, the number of pregnancies was not associated with SRB. In women with 2 parities, the number of pregnancies was poorly associated with SRB, and the ORs were approximately 1.11. Compared with primipara with the first pregnancy, women with three or more parities were 1.39 (95% CI 1.34, 1.43) times more likely to give birth to a boy.

**Table 4:** Association between the number of pregnancy and parity and SRBs in China (2012–2015).

		Parity		
		1	2	≥3
Crude odds ratio*†				
**Number of pregnancies**	1	1	-	-
	2	0.99 (0.99,1.01)	1.11 (1.10,1.13)	-
	≥3	1.01 (0.99,1.02)	1.10 (1.09,1.11)	1.36 (1.32,1.40)
Adjusted odds ratio*†‡				
**Number of pregnancies**	1	1	-	-
	2	1.00 (0.99,1.01)	1.12 (1.10,1.13)	-
	≥3	1.01 (1.00,1.02)	1.11 (1.10,1.12)	1.39 (1.34,1.43)

* Adjusted for the sampling distribution of the population

†Adjusted for clustering of births within hospitals.

‡Adjusted for factors including area, maternal education and mother’s age.

## Discussion

Generally speaking, Asians countries such as Vietnam, India, Korea, and China have relatively high SRB[2, 5–8]. But the SRB in China began to rise earlier, has lasted longer, and has increased by a greater margin. National census and population sampling investigations have shown that the SRB in China has experienced a 30-year-long imbalance since the 1980s, starting with a slight increase in 1982 (108.94), and reaching a record high of 120.18 in 2004. Starting in 2008, however, China’s SRB fell for seven consecutive years after more than twenty years’ increase. Despite the decline, however, the SRB in 2015 stayed at 113.51, still far higher than the normal value (103–107) (S1 Fig).

Based on the data of national census and population sampling investigation, it was estimated that by 2020 men would outnumber women by 30 million or more [28]. Such an estimate has aroused great concern from both the Chinese government and among ordinary people across the nation because SRB is closely related to sex balance and family structure, and to social harmony and stability. Moreover, sex-selective abortion related to high SRB directly affects the survival right of the fetus.

Compared with officially reported SRBs, the SRB estimated from hospital-based data are more reliable. In our study, the monitored SRB was higher than the average SRB in a large population but lower than the SRB obtained from national census or population sampling investigations (S1 Fig). This indicates that there are indeed more baby boys than baby girls in China, but the SRB is not as serious as warned in official reports. This may also indicate indirectly that the SRB obtained from population-based national census and sampling investigation may be unrealistically high.

We also found that the SRB showed a decreasing trend from 2012 to 2014 but increased slightly again in 2015. The increasing trend in the 2015 disparity may be attributed to the belief that babies, esp. girls, born in the Year of the Goat would bear more hardships later in life[29]. 2015 was the Year of the Goat; therefore, many parents-to-be chose to postpone pregnancies[30], which accounted for the SRB rebound in 2015 from that of 2014. In fact, the steady decline in China’s SRB may result from a series of laws and regulations, and intervention measures that forbid sex selection of the fetus, advocate care for girls, and establish a complete social security system, including “The Maternal and Infant Healthcare Law of The People's Republic of China” and the “Population and Family Planning Law of The People’s Republic of China,” which both aim to protect maternal and infant rights[2], as well as “The Plan for Development of Chinese Women” and “The Plan for Development of Chinese Children”.

The SRB in China varies by region and population and differs even within the same region and among the same group of people. A higher SRB has been found in eastern China than in central and western China[9, 31], but SRB differences between the urban and rural areas within a certain region have not been reported. In the present study, we found that the SRBs of rural areas are higher than those of urban areas in eastern and western China. The SRB of urban areas in central China is practically the same as that in rural areas. Families of rural areas in both eastern and western China prefer baby boys, but such a preference has developed out of different backgrounds. In eastern China whose economy is the most developed in the country, clan culture still exits[32], and the core value of clan culture is patrilineal descent. This culture results in a strong son-preference to carry the family name. By contrast, rural areas in the western China are the most undeveloped. In these areas, the incomplete social security system and extreme poverty have forged the tradition of “raising sons to provide for old age” because daughters are supposed to move to the husband’s family upon marriage[33]. Tragically, this tradition is sustained by the convenience of modern fast and safe sex-identification techniques like ultrasound B [2,4,9] and chromosome examination[34]. Wang XF reported that the abortion rate of 441 fetuses is 56.7% for baby girls and 43.3% for baby boys, but the situation is even worse when the existing children are all girls, with the abortion rate being far higher for baby girls (74.8%) than for baby boys (25.2%)[35].

It has been reported that SRB is inversely related to the number of parities under normal sex selection[36–38]. Nevertheless, our study shows that SRB increases as the number of pregnancies and parities increases, and that the margin of increase differs immensely from the increase between the rural and urban areas and among different regions. Of the more than 5 million live births in our study, 62% are primiparity babies. The primiparity SRB remains within or close to the normal range, regardless of the number of pregnancies. The SRBs of parity 2 and parity 3 and above were higher than the normal value. By contrast, a study of 2,000,812 births in 33 countries of sub-Saharan Africa shows that in the group of 20–39-year-old women, the SRB significantly declines with increasing birth order[37]. Another study[38] indicates that a slight decline in sex ratio is associated independently and significantly with birth order. Unlike many other countries where SRB declines with birth order, the sex selection in China mainly starts with the second child. After having the first child, parents who desire a son tend to select the sex of the baby through multiple pregnancies and parities. Although the one-child policy has been the basic national policy of China in theory, a “one-and-a-half-child policy” is the acquiescence in rural areas. Under this policy, parents whose first child is a girl are allowed to have a second child four years after the birth of the first child but they are not allowed to have a third child whether the second child is a boy or a girl[39]. To some extent, such a policy prevailing in rural areas has actually encouraged sex selection[40]. Moreover, when the “one-and-a-half-child policy” fails in giving the family the boy desired, they usually try to continue pregnancies until the birth of a boy, ignoring the family planning policy. That is why most rural families have two or more children, and even 5 to 6 children[41]. In 2015, China terminates its one-child policy that has lasted for over 30 years, and a two-child policy has taken over. It is estimated that China will have 2 to 4 million more babies annually after the two-child policy is implemented [42]. The Health and Family Planning Commission of the People's Republic of China predicted that in the first several years of the policy, the proportion of elder primipara may increase[43]. In the present study we found that an older maternal age is related to a higher SRB. It seems that sex preference is stronger among older women because their chances of pregnancy decrease with age. Therefore, the two-child policy may be a choice for parents with sex preference. Nevertheless, the impact of sex selection on the SRB still needs further observation and research.

Two limitations must be considered in interpreting our findings. First, the NMNMSS over-sampled large referral facilities were all located in urban areas, but we did not know the distribution of hospitals performing deliveries; we therefore only weighed the data by the population distribution for each of the sampling strata. Second, because the sex of previous birth(s) for non-primipara in NMNMSS was unknown, we did not explore the association between the sex of previous birth(s) and the sex of the most recent birth.

## Conclusions

The SRB obtained from hospital-based research shows that more boys have been born than girls in China. Although such a ratio is lower than that obtained from the population-based national census and population sampling investigation, it is objective and truthful. The high SRB in China may have resulted from sex selection. To be more specific, son-preference is inversely related to the educational level and positively related to the age and number of pregnancies. Therefore, under the universal two-child policy, the government should strengthen supervision of son-preference and prevent a dynamic change in SRB.

## Supporting Information

S1 Table Distribution of the National Maternal Near Miss Surveillance System (NMNMSS) in China * Source: Census 2010. (DOCX)

S1 Fig The time trends of SRB using data from national data and the NMNMS. (TIF)
