# Supplementary material for: The Sex Ratio at Birth for 5,338,853 Deliveries in China from 2012 to 2015: A Facility-Based Study
Source: PLoS One. 2016 Dec 12;11(12):e0167575. doi: 10.1371/journal.pone.0167575 (PMC5152891; doi:10.1371/journal.pone.0167575)
Supplement: S1 Table — (DOCX) [file pone.0167575.s001.docx]

**S1 Table**

| **Stratification** | | **Population (%) in NMNMSS in 2010** | **Population (%) in China in 2010*** |
| --- | --- | --- | --- |
| Eastern region | | 66,452,064 (32.5) | 469,411,807 (35.2) |
|  | Rural | 25,844,418 (12.6) | 237,042,702 (17.8) |
|  | Urban | 40,607,646 (19.9) | 232,369,105 (17.4) |
| Central region | | 76,466,774 (37.4) | 503,041,295 (37.8) |
|  | Rural | 43,005,860 (21.0) | 355,482,157 (26.7) |
|  | Urban | 33,460,914 (16.4) | 147,559,138 (11.1) |
| Western region | | 61,434,006 (30.1) | 360,357,767 (27.0) |
|  | Rural | 25,962,225 (12.7) | 238,583,230 (17.9) |
|  | Urban | 35,471,781 (17.4) | 121,774,537 (9.1) |
| All |  | 204,352,844 (100.0) | 1,332,810,869 (100.0) |
