# Supplementary figures and images for: The Sex Ratio at Birth for 5,338,853 Deliveries in China from 2012 to 2015: A Facility-Based Study
Source: PLoS One. 2016 Dec 12;11(12):e0167575. doi: 10.1371/journal.pone.0167575 (PMC5152891; doi:10.1371/journal.pone.0167575)

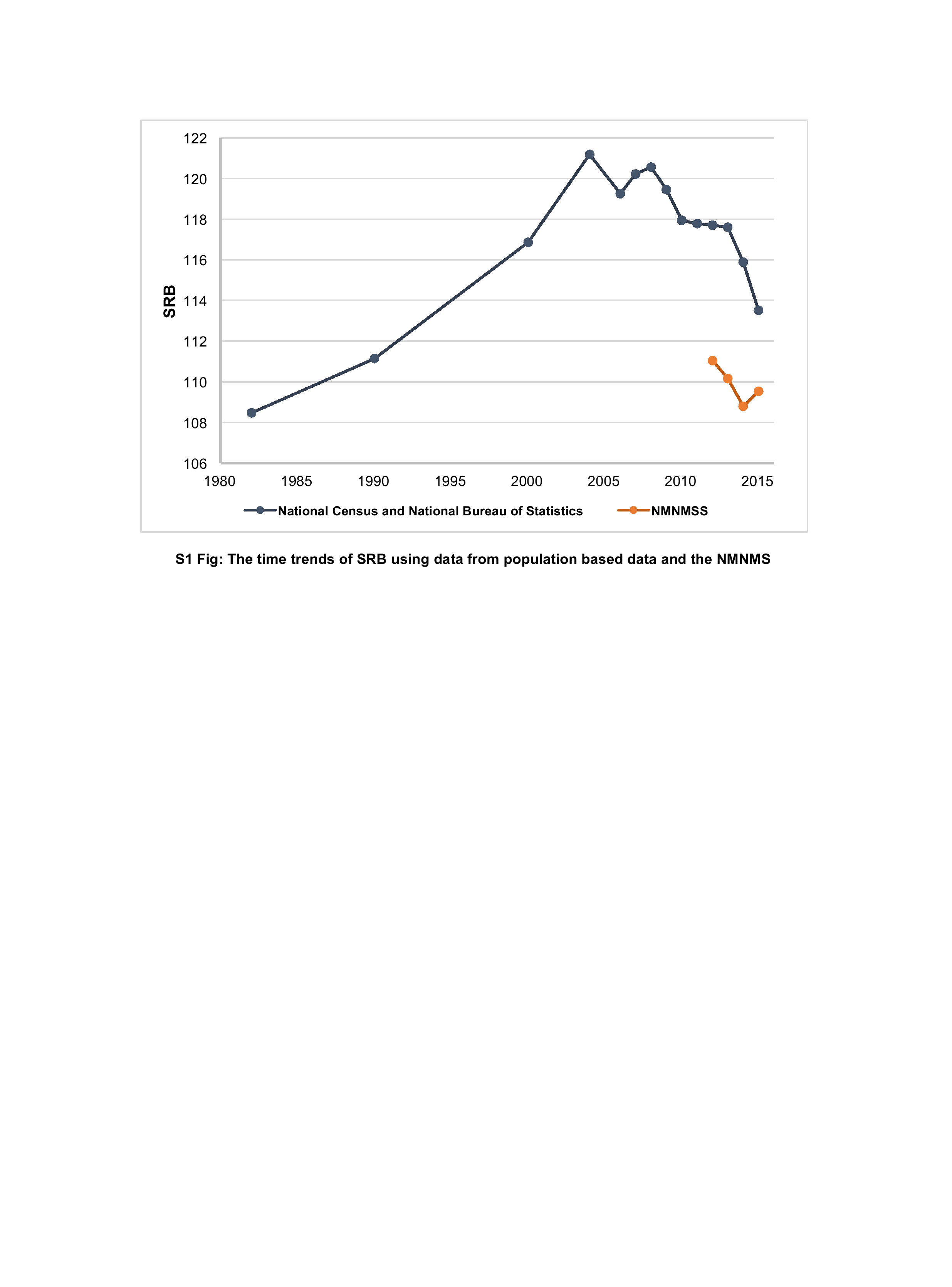

Supplement: S1 Fig — (TIF) [file pone.0167575.s002.tif]
